# Independent Evaluation of the integrated Community Case Management of Childhood Illness Strategy in Malawi Using a National Evaluation Platform Design

**DOI:** 10.4269/ajtmh.15-0584

**Published:** 2016-03-02

**Authors:** Agbessi Amouzou, Mercy Kanyuka, Elizabeth Hazel, Rebecca Heidkamp, Andrew Marsh, Tiope Mleme, Spy Munthali, Lois Park, Benjamin Banda, Lawrence H. Moulton, Robert E. Black, Kenneth Hill, Jamie Perin, Cesar G. Victora, Jennifer Bryce

**Affiliations:** Institute for International Programs, Johns Hopkins Bloomberg School of Public Health, Baltimore, Maryland; National Statistical Office, Zomba, Malawi; Chancellor College, University of Malawi, Zomba, Malawi; University of Pelotas, Pelotas, Brazil

## Abstract

We evaluated the impact of integrated community case management of childhood illness (iCCM) on careseeking for childhood illness and child mortality in Malawi, using a National Evaluation Platform dose-response design with 27 districts as units of analysis. “Dose” variables included density of iCCM providers, drug availability, and supervision, measured through a cross-sectional cellular telephone survey of all iCCM-trained providers. “Response” variables were changes between 2010 and 2014 in careseeking and mortality in children aged 2–59 months, measured through household surveys. iCCM implementation strength was not associated with changes in careseeking or mortality. There were fewer than one iCCM-ready provider per 1,000 under-five children per district. About 70% of sick children were taken outside the home for care in both 2010 and 2014. Careseeking from iCCM providers increased over time from about 2% to 10%; careseeking from other providers fell by a similar amount. Likely contributors to the failure to find impact include low density of iCCM providers, geographic targeting of iCCM to “hard-to-reach” areas although women did not identify distance from a provider as a barrier to health care, and displacement of facility careseeking by iCCM careseeking. This suggests that targeting iCCM solely based on geographic barriers may need to be reconsidered.

## Introduction

The Millennium Development Goals (MDGs) threw a spotlight on the need to accelerate progress of women and children's health.^1^ The fourth MDG (MDG4) called for a reduction of two-thirds in child mortality between 1990 and 2015. A landmark publication in 2003 demonstrated that high proportions of child deaths were attributable to causes for which simple and cost-effective interventions were available and recommended that child survival programs should be refocused on evidence-based,^2^ high-impact interventions, delivered through strategies effective in reaching large populations of women and children.^3^ One such strategy is the integrated Community Case Management (iCCM). iCCM is endorsed by the World Health Organization (WHO) and United Nations Children's Fund (UNICEF)^4^–^6^ as a strategy to extend the provision of correct treatment of childhood pneumonia, diarrhea, and malaria beyond health facilities so that more children have access to lifesaving treatments.^7^ Despite important progress, these three infectious diseases still account for 31% of deaths in children under 5 years of age.^8^ Many countries with high rates of under-five mortality have adopted iCCM as a policy, particularly in sub-Saharan Africa.^9^,^10^

The interventions delivered through iCCM are efficacious in reducing deaths from pneumonia through prompt treatment with antibiotics, diarrhea through the administration of oral rehydration salts (ORS) solution and zinc, and malaria through artemisinin-based combination therapy (ACT)—either presumptively or after administration of a rapid diagnostic test to confirm the presence of malaria infection.^4^ However, little is known about the effectiveness of the strategy in reducing under-five mortality when implemented at scale by governments and partners. There is a large and growing body of research focusing on iCCM implementation and intermediate outcomes,^11^ including the iCCM policy adoption process,^12^ the extent and challenges of implementing iCCM in low- and middle-income countries,^9^ the quality of care provided by health-care workers trained in iCCM,^13^,^14^ and the health system supports needed to implement iCCM effectively.^15^

In 2008, Malawi was one of the first countries in sub-Saharan Africa to adopt the iCCM strategy and plan for its implementation nationwide, targeting areas of each district with limited access to fixed health facilities. Located in east Africa, with a population of about 16 million in 2012, Malawi is one the poorest countries in world. It had a gross domestic product per capita of US$815 in 2014,^16^ and ranked 174th of 187 on the human development index in that year.^17^ Despite these daunting statistics, Malawi is among the few countries in sub-Saharan Africa that has achieved MDG4, reducing its under-five mortality rate from 245 deaths per 1,000 live births in 1990 to 68 deaths per 1,000 live births in 2013.^18^ Among the estimated 41,000 under-five deaths in Malawi in 2013, about half (48%) were attributed to infectious diseases, including pneumonia (13%), malaria (15%), diarrhea (8%), and HIV/AIDS (12%).^8^

This article reports on a prospective evaluation of iCCM in Malawi, including assessments of iCCM implementation strength, utilization, costs, intervention coverage, and impact on child mortality.

## Methods

### Description of the iCCM program in Malawi.

The Ministry of Health (MOH) has been implementing iCCM at scale in its 28 districts as part of the national Essential Health Package^19^ since 2009, with support from various partners. The MOH intended that iCCM services at community level would complement the rollout of the Integrated Management of Childhood Illness (IMCI) strategy in first-level health facilities, resulting in rapid increases in coverage for all sick children. Paid government health workers (called health surveillance assistants [HSAs]) are responsible for providing iCCM services to defined target populations of about 1,000 individuals in “hard-to-reach areas,” defined by each district based on geographic access to a fixed health facility (> 8 km or other geographic barrier). As of July 2013, the MOH reported that 3,392 HSAs were providing iCCM services among 9,555 HSAs in the country.^20^

HSAs participated in a 6-day training course on iCCM that adhered closely to the standard WHO/UNICEF iCCM training curriculum.^21^ After training, HSAs received an initial supply of drugs, which they could replenish at no cost from their assigned fixed health facility. Members of the District Health Management Team, including some who had not been trained in correct clinical management of childhood illness, supervised the HSAs initially. Beginning in 2011, some HSAs were provided with clinical mentoring when they visited the fixed health facility to collect their monthly salary and drug resupplies. HSAs are expected to live in their catchment areas and provide child health-care services through at least two special sessions each week and on-demand for sick children brought to them for care. iCCM services and drugs are provided free of charge. Further information on how the iCCM strategy is being implemented in Malawi is available elsewhere.^22^

### Evaluation objectives and impact model.

The objective of the evaluation was to assess the extent to which the introduction of iCCM in Malawi was associated with increases in careseeking for childhood illness, leading to accelerated declines in under-five mortality.

The original design, developed in a workshop conducted in-country in December 2008, was a pretest–posttest quasi-experimental design with six intervention districts selected from among those in which iCCM implementation was supported by WHO and UNICEF under the Canadian-led Catalytic Initiative to Save a Million Lives^23^ (CI) and six comparison districts. However, by 2009, the MOH had secured financial and technical assistance to scale-up iCCM using similar approaches in all districts in the country, precluding the use of an intervention-comparison design.

This and similar developments in other Catalytic Initiative countries prompted the development of a new approach to evaluating programs at scale, called the National Evaluation Platform (NEP).^24^ The NEP uses districts as the unit of analysis and supports various types of analysis. For the impact evaluation of iCCM in Malawi, we used a “dose–response” analysis with measures of iCCM implementation strength as the dose and measures of outcomes (treatment coverage as reflected in careseeking for childhood illness and intervention coverage) and impact (child mortality) as the response, adjusting for appropriate confounders. The NEP design covers 27 of the 28 districts in Malawi. (The 28th district, Likoma, is an 18 km^2^ island in Lake Malawi with an estimated population in 2008 of 10,445 inhabitants.^25^)

The evaluation was designed to test the assumptions underlying a five-step impact model: 1) it is possible to train, deploy, supply with drugs, and supervise a substantial number of HSAs who will provide iCCM for malaria, pneumonia, and diarrhea (provision); 2) HSAs will be able to provide appropriate, high-quality iCCM services (quality); 3) mothers will take their sick children to HSAs for care (utilization); 4) the proportion of sick children who need care from a trained provider who actually receive that care will increase (coverage); and 5) rates of child mortality will decline (impact).

### Data sources and variables.

Table 1 lists all variables used in the analysis and their sources.

Our original design included prospective collection of routine data related to the implementation of all maternal, newborn, and child health (MNCH) programs at district level. The National Statistical Office (NSO) trained Health Management Information System (HMIS) officers from the 16 districts originally included in the evaluation (10 CI and six non-CI) to coordinate quarterly data extraction using a predesigned tool. Between 2011 and 2013, we abstracted MNCH data from the HMIS and iCCM monthly reporting forms on a quarterly basis in each of these districts. NSO visited each district on a biannual basis to collect the forms and conduct interviews with the District Health Management Team (DHMT)'s staff on contextual factors that could affect MNCH (epidemics, food shortages, vaccination campaigns, etc.). Despite repeated efforts to improve data completeness and quality (e.g., frequent follow-up by phone and in person, refresher trainings, increased per diems, data review meetings, and involving other district staff such as the district health officer and program coordinators), the MOH, implementing partners and the evaluation team agreed in 2013 that the information collected was not sufficiently comprehensive and accurate to be used as the basis for assessing iCCM implementation strength over this period. Important data needed to assess the strength of iCCM implementation were not available in district records (e.g., supervision frequency, stockouts of iCCM drugs and other commodities). Even for data that were available, there were numerous inconsistencies identified through triangulation with MOH records.

In collaboration with the MOH, we therefore developed and tested an alternative approach for collecting data on the strength of iCCM implementation. This method used cellphone interviews to collect data directly from a random sample of the HSAs. The standardized interview protocol asked HSAs for information on a core set of iCCM implementation strength indicators (supervision, training, utilization, drug stocks, etc.) that had been agreed upon by the MOH and iCCM implementation partners, which in turn reflected global consensus benchmark indicators for iCCM.^26^ In the validation study,^27^ the research team visited each interviewed HSA within 48 hours to confirm their response via records at their assigned fixed health facility and inspection visits at the HSA's village clinic. The sensitivity and specificity of the measurements collected via cell phone interviews were above 80% for all indicators, and many were higher (> 90%).

We therefore proceeded to implement the “implementation strength snapshot” method among a census of all iCCM-trained HSAs in the country in mid-2013. The MOH reported that the iCCM program was being implemented at full strength at this time, although delays in the endline household survey created a gap of several months between the measurements of implementation strength and intervention coverage and mortality. Further details on the methods of this iCCM implementation strength snapshot are available elsewhere^20^ and in Supplemental Web Annex, Part 1.

We used provision of iCCM at the district level as measured through the implementation strength snapshot as the dose variable, and defined it as the density of iCCM-ready HSAs per 1,000 under-five population, as shown in Table 1. We estimated the utilization of iCCM services by asking each iCCM-trained HSA how many sick children he/she had managed in the previous month. We used population data to translate this estimate into the estimated number of sick children treated by HSAs per 10,000 under-five population in the previous month, and child contacts with an iCCM-trained HSA per child per year.

We used data from household surveys conducted by the Malawi NSO in 2010 and 2014 to estimate levels of careseeking for childhood illnesses addressed by iCCM and child mortality between the ages of 2 and 59 months, which were the outcome and impact indicators, respectively. We considered both as “response” variables in the analysis. The 2010 survey was conducted under the Demographic and Health Surveys (DHS) program,^28^ and the 2014 MDG survey was conducted under the UNICEF-supported Multiple Indicator Cluster Survey (MICS) program.^29^ The surveys used comparable methods and interviewed samples of about 1,000 households representative of each district. The clustered sample design and sample weights were taken into account when calculating district-level estimates. The two surveys had similar questions on careseeking for illness and included HSA or village worker/HSA as a specific response code.

We used careseeking as a proxy for treatment coverage for all three infectious diseases targeted by iCCM. The careseeking data were reanalyzed by the study team to ensure consistent definition across the two surveys. We defined “formal providers” as either public (government) or private health facilities or community-based workers (e.g., hospitals, clinics or mobile clinics, physicians, nurses, and HSAs). Private pharmacies, shops/vendors, traditional practitioners, and non-health system sources (friend/neighbor) were not considered formal providers.

In the main analyses, we report on careseeking for all three conditions (pneumonia, diarrhea, and malaria) combined for children aged 2–59 months. Results for individual conditions and by type of provider are presented in Supplemental Web Annex, Part 2. In 2010, the survey also included a question on geographic access to services, asking mothers: “Is distance a problem for obtaining health care?” We used the responses (yes/no) as a stratifier in reporting the careseeking results at baseline. Although this question referred to the woman's own health care, we considered it as an adequate proxy for perceived barriers to careseeking for family health. This question was not included in the 2014 survey.

During the surveys, trained fieldworkers obtained full birth histories from women of reproductive age to estimate child mortality. We conducted the analysis using changes in mortality for both all under-five children and children aged 2–59 months, as the latter group is the specific target of iCCM as implemented to date in Malawi. There were no differences in the results for these two age groups, and we therefore present results on deaths among children aged 2–59 months in the article and the under-five mortality results in Supplemental Web Annex, Part 3. We conferred with the MOH to define the pre-iCCM (baseline) period as October 2007 to September 2009, and the full iCCM implementation period (endline) as October 2010 to September 2013, for the purpose of mortality analysis. Data on deaths from the iCCM phase-in period of October 2009 to September 2010 were not included in the analyses.

We anticipated that both the density of health facilities in a district and the density of health facility workers could be confounders in the analysis. The IMCI unit within the MOH therefore contacted all districts in November 2014 to obtain information on the numbers of health facilities and health facility workers, and the results were included in the analyses as potential confounders.

The analyses of inequalities in careseeking included stratification by urban/rural residence and wealth quintiles, as measured in the 2010 and 2014 household surveys. For the latter, we used asset indices based on household possessions and building materials, calculated with the same methodology in the 2010 and 2014 surveys.^30^

We estimated the economic costs of providing iCCM through HSAs in 2012 U.S. dollars. Data sources included HSAs, health facilities, district health offices, and implementing partners. We collected data on HSA salaries, equipment, drugs, training, supervision, and other program costs. We estimated cost per HSA, cost per district, cost per child, and the total cost of the iCCM program in 2012. Details of the costing methods and results are available in Supplemental Web Annex, Part 9.

### Plan of analysis.

We used an NEP approach, including dose–response analyses of iCCM implementation strength on the defined outcomes across 27 of the 28 districts in the country.

We produced descriptive statistics for all variables at district level and assessed correlations among them.

We estimated careseeking for each district at baseline using the 2010 DHS. We defined the careseeking variable as the percent of children aged 2–59 months reported by their caregiver to have had diarrhea, pneumonia, or fever in the past 2 weeks, who received care from a formal provider. For the same reference group of children, we also estimated the percent receiving care from a formal provider at endline for each district from the 2014 MICS survey. We use the difference in these estimated percents for each district as our outcome of interest. We also estimated the mortality rates among children aged 2–59 months separately for baseline and endline in each district, using full birth histories from the 2014 MICS survey. We used the difference between these mortality rates as another outcome measure. We conducted ordinary least squares (OLS) regression analyses relating iCCM implementation strength (the dose) with changes in careseeking and mortality rates (the responses). We then used multiple linear regression to examine the relationship between the dose and the two response variables, adjusting for district population and the density of health facilities and health facility workers. We also carried out dose–response analyses using more complex, two-stage least squares (TSLS) models; the results were similar, so we report only the OLS results here. The TSLS results are available in Supplemental Web Annex, Part 4.

We used two approaches to take baseline levels of the outcomes into account. The first was to incorporate changes in the levels of the outcome variables (careseeking, mortality) in the analyses as change variables (endline minus baseline levels); these analyses are presented here. We did not attempt to use relative changes as response variables, because this would not affect the overall results. We also used the endline value as the dependent variable, including the baseline value as one of the independent variables; the results of this analysis are available in Supplemental Web Annex, Part 5.

iCCM was designed as an intervention for rural areas, so we explored the possibility of restricting all analyses to rural areas. However, the 2014 MDG survey indicated that 86% of the Malawi population is rural, and only two districts (Blantyre and Lilongwe) are more than 30% urban. We repeated the main analyses after excluding these two districts and found that associations with iCCM implementation strength were virtually unchanged. We therefore present results only for the full population in the 27 districts.

### Role of the funding source.

The sponsors had no role in the analysis and interpretation of the evidence, in writing the paper, or in the decision to submit for publication. All authors, including the corresponding author, had full access to all the data and participated in the decision to submit the manuscript for publication.

## Results

**Figure 1.**
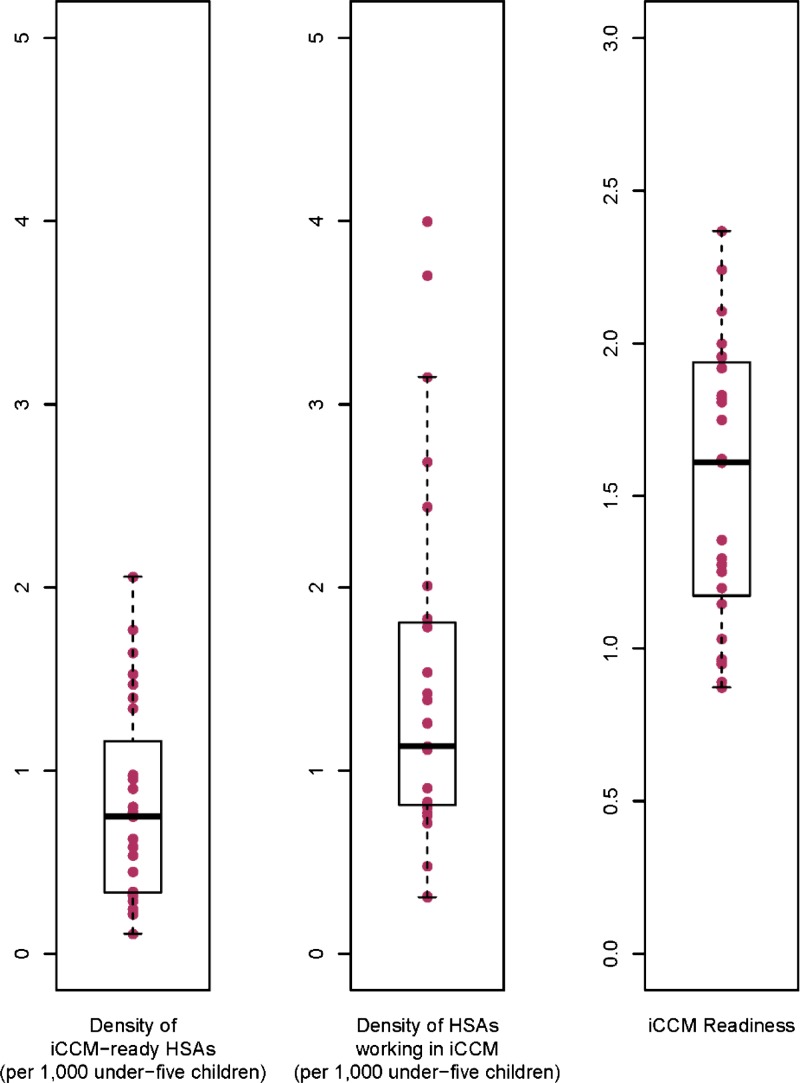
District medians and spread for three component measures of iCCM implementation strength in Malawi (*N* = 27 districts). HSAs = health surveillance assistants; iCCM = integrated Community Case Management.

Table 2 shows the means, medians, and ranges of all study variables and the year in which data were collected for each. Figure 1

shows the district medians and spread for the three components of the iCCM implementation strength measure.

The results of the 2013 census of iCCM-trained HSAs show that 3,392 HSAs were trained in iCCM and on average 1.5 HSAs per 1,000 under-five population were actively managing sick children at that time.^20^ The findings indicate that levels of iCCM system support for the HSAs providing iCCM were moderate: 58% of HSAs reported they were supervised in the previous 3 months with reinforcement of clinical practice and 59% reported no stockouts of key iCCM drugs (co-trimoxazole, lumefantrine–artemether, ORS, and zinc) in the previous 3 months lasting longer than 7 days.^20^

Districts showed substantial variability in all indicators. On average, there were fewer than one iCCM-ready HSA per 1,000 children under five per district (range: 0.1–2.1), and utilization was 778 children treated per 10,000 children under five, or about 0.93 contacts per child per year. The under-five mortality rate dropped by about 30% in the evaluation period. Nationally, careseeking for pneumonia, diarrhea, and malaria (combined) remained stable at 70% at baseline and endline (Table 2). Careseeking from HSAs increased from 2% to 10%. Careseeking results for individual diseases and by type of provider are available in Supplemental Webannex, Part 2. Of women, 57% reported that distance to a health facility was a problem in accessing health care in 2010 (range: 37–81%).

We correlated all indicators in Table 2 among themselves, with the 27 districts as the units of analysis. The full correlation matrix is available in Supplemental Web Annex, Part 6. Selected results of particular interest that were significant at the *P* < 0.05 level include those showing that higher district under-five population was associated with lower rates of utilization of HSAs; higher HSA density and higher density of iCCM-ready HSAs were associated with higher HSA utilization rates; and higher levels of careseeking for childhood illness at baseline were associated with smaller district populations and higher levels of maternal schooling and densities of heath facility workers. Baseline mortality was not related to any of the contextual factors, and neither the iCCM implementation strength indicator nor its component parts were correlated with changes in careseeking or mortality. Mothers' perceptions that distance was a problem in accessing health care at baseline were not associated with larger increases in careseeking from an HSA between 2010 and 2014.

Figure 2

**Figure 2.**
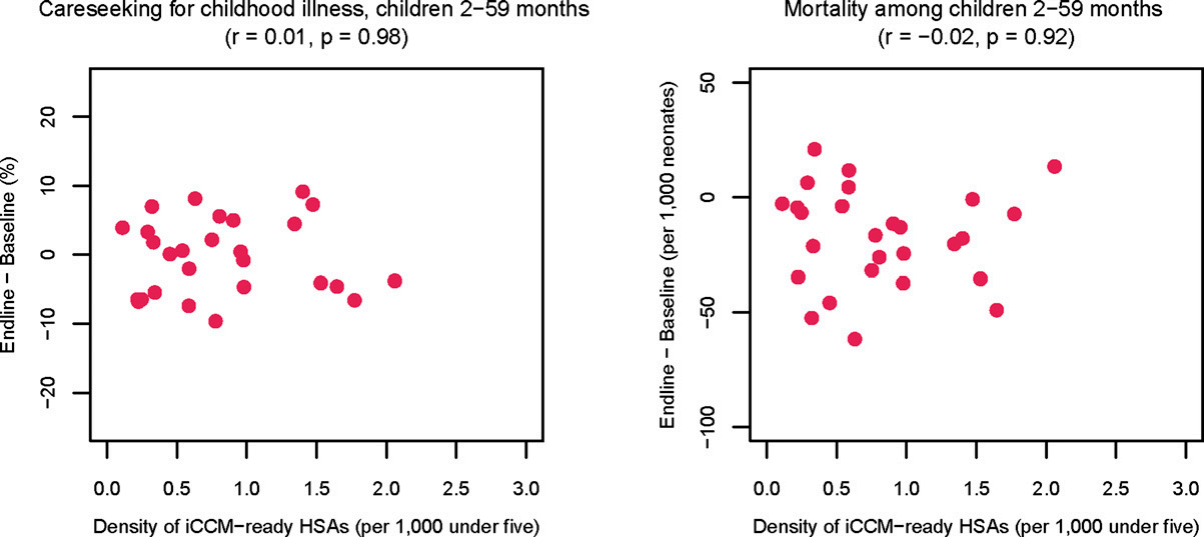
Correlations between the density of iCCM-ready HSAs and changes between 2010 and 2014 careseeking and mortality for children aged 2–59 months in Malawi (*N* = 27 districts). HSAs = health surveillance assistants; iCCM = integrated Community Case Management.

shows a lack of association at district level between our primary dose variable reflecting the density of iCCM-ready HSAs and either of our two response variables: careseeking for childhood illness and mortality. Neither of the component parts of the dose measure—density or readiness—were associated with changes in careseeking for childhood illness or mortality; additional results are available in Supplemental Web Annex, Part 7.

Table 3 shows the results of OLS linear regressions of change in mortality and careseeking, adjusted for confounding district population, density of health facility workers, and density of iCCM-ready HSAs based on the bivariate correlations shown in Supplemental Web Annex, Part 6. Neither effect is statistically different from zero, and both are of small magnitude. We examined diagnostics for these analyses, both for the change in district careseeking and for the change in district child mortality rates. For these regression results to be valid, the residual change must approximate a normal distribution. In a Shapiro–Wilk test for normality,^31^ we found no evidence that the change in careseeking (*P* = 0.49) or the change in mortality (*P* = 0.52) was contrary to this assumption.

Figure 3

**Figure 3.**
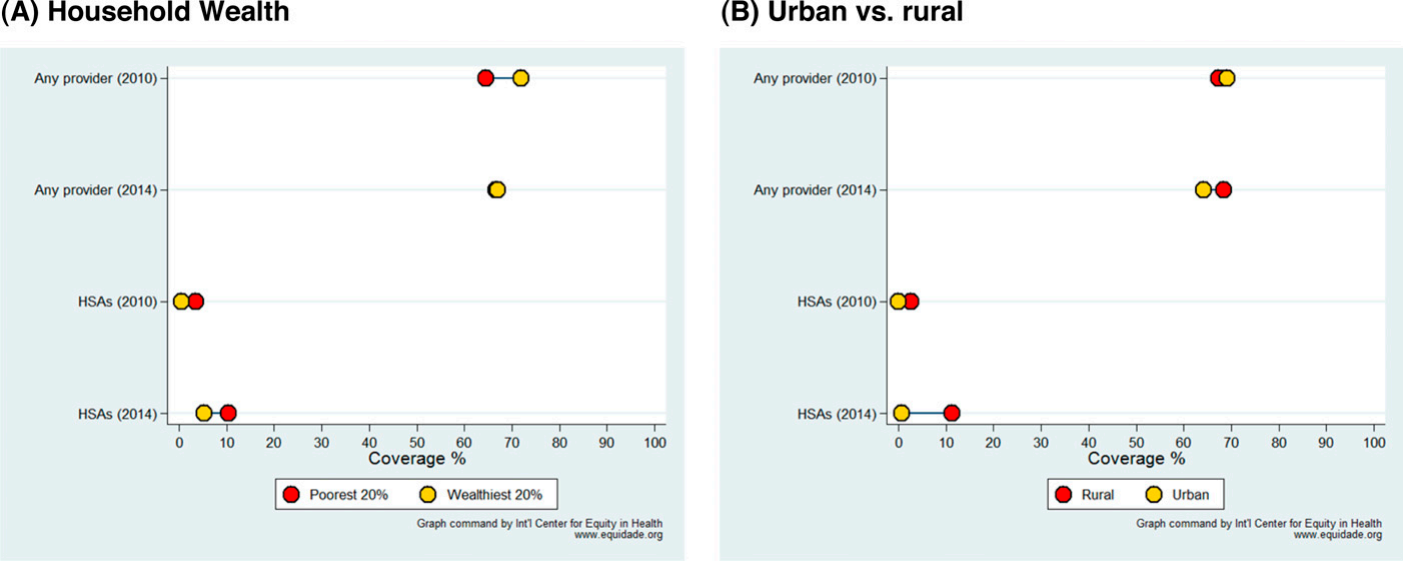
Careseeking for childhood illness in 2010 and 2014among children living in (**A**) the poorest and wealthiest quintiles of the population and (**B**) households in rural and urban areas, Malawi. HSAs = health surveillance assistants

shows careseeking for childhood illness disaggregated by household wealth (poorest and richest quintiles) and between rural and urban households. Figure 3A shows that in 2010, careseeking from any provider was about 10% points higher in the wealthiest quintile than among the poorest. By 2014, this difference had disappeared, but this was mostly due to a drop in careseeking among the rich. Careseeking from HSAs increased over time, particularly for the poorest quintile. By 2014, HSAs were managing 10.4% of all episodes in this group. Careseeking from other providers fell by approximately the same extent as HSA careseeking increased, in all wealth groups (Supplemental Web Annex, Part 8).

Figure 3B shows that careseeking rates were virtually identical in 2010 for urban and rural children. By 2014, rural children had a slight advantage of about 4 percentage points. HSAs were appropriately focusing on their work in rural areas, and HSAs saw virtually no urban children. Careseeking from HSAs increased from 2.5% to 11.1% of all episodes among rural children between 2010 and 2014. In 2010, caretakers answered a question on whether they considered distance as a problem in accessing health care. For those answering “yes,” the careseeking rate was 66.3%, compared with 69.7% for those who answered “no” (*P* = 0.06).

The average annual cost of providing iCCM was US$1,812 (95% confidence interval [CI]: $1,327–$2,304) per HSA with an average cost per case seen of US$1.86 (95% CI: $1.36–$2.43). The total cost of the national program in 2012 was estimated at US$6.96 million (95% CI: $5.10–$8.85), with HSAs' salaries and drugs accounting for 52% and 28% of the total, respectively. This corresponds to an annual cost of US$2.43 per under-five child per year (95% CI: $1.78–$3.09) (Table 4).

## Discussion

This first NEP analysis of the effects of the national scale-up of the iCCM strategy in Malawi found no relationship at district level between the strength of iCCM implementation and changes between 2010 and 2014 in careseeking for childhood illness or mortality. We examined possible reasons for this by returning to the impact model underlying the evaluation design and using it to interpret our findings and related evidence on iCCM provision, quality, utilization, coverage, and impact. All findings have been reviewed and discussed with program implementers including the MOH and are being used now to plan for strengthened MNCH and nutrition programs in Malawi.

In terms of provision, Malawi has demonstrated that it is possible to train, deploy, supply with drugs, and supervise a substantial number of HSAs who will provide iCCM for childhood pneumonia, diarrhea, and malaria. As of mid-2014, the MOH and its development partners have provided high-quality iCCM training to about 3,500 HSAs, or 1.5 iCCM-trained and active HSAs per 1,000 under-five children (H. Nsona, personal communication, November 17, 2014). On the basis of an assumption that on average, the percent of the total population that is under 5 years of age is 17%, this translates into a ratio of one HSA per 5,882 population, well below the MOH target of one HSA per 1,000 population. Among those trained, about six in 10 reported in 2013 that their clinical skills in iCCM had been reinforced by a supervisor or mentor within the last 3 months, and a similar proportion reported continuous supplies of all essential iCCM drugs for the same period. Despite these considerable achievements, there is clearly room for improvement in the provision of iCCM services.

The MOH in Malawi deploys iCCM-trained HSAs to district-defined areas with limited geographic access to fixed health facilities, or hard-to-reach areas. We considered restricting the analyses to such areas, but despite repeated attempts, it was not possible to obtain precise boundaries from implementing partners. In addition, iCCM was a district-based strategy that included health systems changes at district level, not only in specific areas. Furthermore, the premise behind the deployment of iCCM in hard-to-reach areas was that careseeking was particularly low in such areas, and therefore careseeking rates at district level would be improved by reaching such children who were previously unreached; our results at district level should have picked up such an increase, had it occurred.

The results reported here suggest that this geographic targeting may need to be re-examined. In 2010, 57% of mothers reported that geographic distance or barriers were a problem in accessing health care, and we found no association between mothers who reported this problem and changes in careseeking between 2010 and 2014, after the introduction of iCCM. What iCCM planners did not know in 2010, when they were planning the rollout of the strategy, was that careseeking for childhood illness had already increased dramatically at that time, from about 50% or less for all three diseases targeted by iCCM in 2006^32^ to 70% in 2010. The results from the 2010 survey were not released until 2011, by which time iCCM was fully rolled out. This illustrates the importance of having sound, recent data available to support MNCH decisions and suggests that geographic access alone cannot explain why iCCM did not result in increases in careseeking for childhood illness. It also suggests, with benefit of hindsight, that one of the key assumptions behind iCCM implementation was not supported by evidence.

Our findings on the density of iCCM-ready HSAs—reported here as an average of 1.5 HSAs providing iCCM per 1,000 under-five children—must be interpreted in context. The MOH target was to train and deploy one HSA for each 1,000 total population. The notion of density assumes that every iCCM-trained HSA is present in the community full-time, and available to provide child health services on demand. In fact, only 70% of iCCM-trained HSAs reported that they resided in their catchment areas in the 2013 iCCM implementation strength snapshot,^20^ and even those living in the community are often called away to perform duties other than iCCM. Discussions about the “right” level of density for iCCM are under way now as a part of Malawi's larger redesign of their MNCH strategy.

In terms of quality, a 2009 observation-based assessment of random samples of iCCM-trained HSAs in six districts in Malawi found that they were providing child health care at reasonably good levels,^13^ at least equivalent to the care provided in first-level health facilities at about the same time.^33^ However, in both settings, only about six in 10 sick children presenting for care were correctly assessed for danger signs and managed for an iCCM illness, and among children needing referral (who are the most likely to die), only about half were referred. We report here on a 2013 assessment of a proxy measure of “readiness” to deliver iCCM as reflected in trained workers who were recently supervised and had continuous supplies of essential iCCM drugs, and find an average score of 1.5 on a 0–3 point scale (range: 0.87–2.37), certainly below what is needed and potentially discouraging utilization. Also related to quality is the fact that the current iCCM program does not address deaths among infants less than 2 months of age, estimated in 2014 to represent just over one-third of all under-five deaths.^34^ The MOH is keenly aware that service quality can drive demand and is an essential prerequisite for iCCM impact.

Even the best child health services cannot save lives unless the population uses them. Our results show that in 2013, each iCCM-trained HSA was managing an average of 55 sick children per month, or nearly two sick children per day. This rate is more than 10 times higher than that reported among iCCM-trained community volunteers in nine districts of Burkina Faso,^35^ and over three times higher than that reported by iCCM-trained health extension workers in Oromia Region, Ethiopia.^36^ This rate of utilization translates into an average of about one sick child contact with an iCCM-trained HSA per child per year, which is difficult to interpret without a true estimate of need.

There was no change in rates of careseeking for childhood illness from any formal provider between 2010 and 2014. The increase in careseeking from HSAs—from 2% in 2010 to 10% in 2014—is in the right direction, but this increase replaced facility attendances rather than adding to them. Our finding that almost all of this increase occurred among poor, rural households is encouraging, but again, in the same group facility use was reduced by five percentage points. We found remarkably small inequalities in careseeking in 2010, either by wealth quintile or by urban/rural residence. This is in line with previous cross-country comparisons showing that Malawi is a relatively equitable country in terms of child health indicators.^37^ Even for the poorest and for rural children, HSAs provided services to only about 10% of the 70% of sick children taken outside the home for care, representing about one in seven sick child contacts with health-care providers. We must gain a better understanding of the characteristics of the children who are not being taken for care and their families, and why they are not being taken for care.

Although HSAs appear to be largely replacing other sources of care, this may be a positive finding if families who are already seeking care are able to find care closer and more conveniently. This should be addressed by further research, which should also investigate why about 30% of HSAs do not reside in the community to which they are allocated and why careseeking rates from HSAs are lower than initially expected. A 2012 study shows that in one district of Malawi, iCCM increased geographic access to health care, but the increase in “effective access”—access to a trained and equipped health-care worker—was much lower, only one-third of the increased geographic access.^38^

The costing results suggest that a case of childhood illness may be treated in the community by an HSA for about US$1.86. Because HSAs are an existing cadre in Malawi whose salaries are already financed through the government, the incremental cost of iCCM may be lower than in other settings. Our results are in line with those reported recently by Collins and others,^39^ except that we identified drugs as the main cost item (see Supplemental Web Annex, Part 9), whereas in their study, salaries were ranked first. An important limitation is that we collected data only on direct costs, and the results therefore do not reflect opportunity costs, such as the difference in costs to caregivers of accessing care in settings close to home.

This study is limited by the “real-world” challenges of evaluating programs being implemented by government at scale. First, use of district populations as the denominator for our implementation strength measure was appropriate given the intention of the program to improve careseeking, coverage, and therefore population health for all children within a district. However, a companion assessment of the impact of iCCM in the hard-to-reach areas targeted by the program would have been useful in interpreting the results. Second, we used careseeking as a proxy for treatment because of issues in measuring treatment coverage.^40^ However, careseeking may not reflect correct treatment, particularly in areas with frequent stockouts. Nevertheless, results based on the careseeking measures are more conservative than true treatment measures, given the former are by definition higher than the latter. Third, our baseline measure for careseeking, based on the 2010 DHS, may have been affected by early iCCM implementation, but only about 2% of all careseeking at that time was received from an HSA (Table 2). Fourth, we measured mortality retrospectively, and the baseline period was October 2007 to September 2009, that is, prior to iCCM implementation. Fifth and finally, our measure of iCCM implementation strength may be too narrow, especially given that the three items in the readiness scale are not independent of one another. However, the measure reflects the core components of the iCCM strategy as defined by the MOH and implementing partners. We conducted the national mobile phone census of HSAs in 2013 using validated methods^18^ at the request of the MOH, after the data provided through partner reporting and the routine information system fell short of what was needed. Nevertheless, measurement of implementation strength is a complex task, and further methodological work is needed. Regardless of the quality of our measurement of implementation strength, however, the undisputed finding that overall careseeking for childhood illness did not increase provides strong evidence that the iCCM program has not yet achieved its original goal.

Our negative results must be interpreted in light of important contextual factors that were present in Malawi. The proportion of children already accessing care at baseline was very high, unlike most countries in sub-Saharan Africa.^41^ There was no association between the proportion of mothers who reported that distance was a problem for access to care and careseeking from an HSA. Under-five mortality had improved markedly during the previous decade, and the decline continued throughout the country during the evaluation, making it more difficult to pick up an additional acceleration in the rate of reduction that might be attributable to iCCM. Nevertheless, the fact that the regression and correlation coefficients derived from the dose–response analyses were virtually equal to zero suggests that lack of statistical power to detect a significant reduction was not a problem. Finally, the iCCM program appears to have prioritized the supply side (training, drugs, etc.) rather than the demand side, through which effective behavior-change initiatives to promote HSA utilization might have led to important gains in careseeking. For this reason, we do not believe that the present results are necessarily generalizable to other programs in the region.

Malawi has experienced an impressive drop in child mortality in the past two decades.^18^ A recent analysis using the Lives Saved Tool^42^ as part of a Countdown to 2015 in-depth country case study in Malawi shows that this mortality reduction is the result of increases in coverage of treatment of diarrhea, pneumonia, and fever; insecticide treated net coverage; and childhood vaccination coverage.^43^ Although this projection showed that treatment of iCCM illnesses has saved lives since 2001, we found no evidence through this study that those increases in treatment coverage were the result of the iCCM program scale-up.

This analysis points to the benefits of a full evaluation using an NEP approach, which incorporates intermediate measures of implementation strength. We agree with the recommendations of a 2013 iCCM evidence review symposium that assessments of processes and inputs are essential complements to assessments of outcomes and impact in the evaluation of programs being delivered at scale by governments and partners.^44^ The public health community must continue to invest in full and rigorous evaluations of such programs, including their impact on population health, as a way to improve the effectiveness of their efforts.

## Supplementary Material

Supplemental Datas.

## Figures and Tables

**Table 1:** Indicators and data sources

Indicator	Definition/notes	Source	Year
Provision and quality (iCCM implementation strength)			
HSA density	Density of HSAs working in iCCM per 1,000 under-five population (trained and treated a child in the previous 3 months)	ISS	2013
iCCM readiness	Summary score ranging from 0 to 3 measured among HSAs who reported managing a sick child in the previous 7 days, based on: 1) receipt of iCCM supervision at their place of work in the community in the previous 3 months; 2) reinforcement of clinical practices (through observation of case management, practicing case scenarios, or mentoring) during most recent supervision; 3) no stockouts of essential iCCM drugs in previous 3 months	ISS	2013
iCCM-ready HSA density	Density of HSAs with high iCCM readiness (readiness score of 2 or 3) per 1,000 under-five population	ISS	2013
Utilization			
iCCM utilization	Sick children treated by HSAs per 10,000 under-five population in previous month	ISS	2013
Coverage (outcome)			
Careseeking for childhood illness from formal health-care providers	Children reported to have suspected pneumonia, diarrhea, or fever/malaria and have been taken for care to a formal provider	DHS	2010
		MDGE	2014
Careseeking for childhood illness from an HSA	Children reported to have suspected pneumonia, diarrhea, or fever/malaria and have been taken for care to an HSA	DHS	2010
		MDGE	2014
Impact			
2–59 months mortality rate	Probability that a child surviving until 1 month will die before reaching 5 years of age, per 1,000 live births, for baseline (2007–2009) and endline (2010–2013) periods	MDGE	2014
Contextual factors			
Under-five population	Population of children under 5 years of age	Census	2008
Total population	Total population	Census	2008
Poverty	% of households living below 2011 Malawi national poverty line	IHS3	2010
Maternal education	% of mothers having any level of education	DHS	2010
Health facility density	Density of health facilities per 10,000 total population	MOH	2014
Health facility worker density	Density of facility worker per 10,000 total population	MOH	2014
Distance to health facility perceived as a problem	Proportion of women who responded that distance to health facility is a problem in accessing health care	DHS	2010

DHS = demographic and health survey; HSA = health surveillance assistant; iCCM = integrated Community Case Management; IHS3 = integrated household survey 3; ISS = implementation strength snapshot; MDGE = millennium development goals endline survey; MOH = Malawi Ministry of Health.

**Table 2:** Unweighted descriptive results for district variables included in the dose–response analysis and year in which data were collected, for 27 districts in Malawi

District variables	Year	Mean	Median	Minimum	Maximum
Provision and quality (iCCM implementation strength)					
HSA density (per 1,000 under-five children)	2013	1.5	1.1	0.3	4.0
Average iCCM readiness score	2013	1.5	1.6	0.9	2.4
iCCM-ready HSA density (per 1,000 under-five children)	2013	0.8	0.8	0.1	2.1
Utilization					
Children treated by HSAs in the previous month (per 10,000 under-five children)	2013	778	776	200	1,524
Coverage					
Baseline careseeking for iCCM conditions	2010	70.2	69.6	58.1	82.6
Endline careseeking for iCCM conditions	2014	69.8	70.4	60.1	77.9
Change in careseeking for iCCM conditions between baseline and endline	–	−0.3	−0.1	−9.3	8.7
Baseline careseeking from HSA for iCCM conditions	2010	2.4	1.9	0.0	6.5
Endline careseeking from HSA for iCCM conditions	2014	10.4	9.1	2.3	23.6
Change in careseeking from HSA for iCCM conditions between baseline and endline	–	8.0	6.9	−0.7	19.5
Impact					
Baseline 2–59 months mortality rate (2007–2009)	–	62.7	55.8	31.2	102.3
Endline 2–59 months mortality rate (2010–2013)	–	45.5	48.1	23.2	69.6
Change in 2–59 months mortality rate between baseline and endline	–	−17.3	−16.5	−61.5	21.0
Contextual factors					
Under-five population (in thousands)	2008	88,089	77,707	16,701	336,695
Poverty (%)	2010	53	46	24	82
Any maternal education (% of mothers)	2010	85	86	65	99
Health facility density (per 10,000 total population)	2014	0.52	0.43	0.18	1.30
Health facility worker density (per 10,000 total population)	2014	13	12	7	26
Proportion of women reporting that distance to health facility is a problem in accessing health care	2010	57	57	37	81

HSA = health surveillance assistants; iCCM = integrated Community Case Management.

**Table 3:** OLS regression of the change in careseeking and mortality among children 2 to 59 months in Malawi, predicted by implementation strength and contextual factors

Outcome	Predictors	Estimate	SE	*P*
Change in careseeking between baseline and endline (% points)	Intercept*	6.83	4.52	0.145
	District population (total population/100,000)	−0.27	0.56	0.627
	Health facility density (per 10,000 total population)	−3.27	5.31	0.544
	Facility worker density (per 10,000 total population)	−0.49	0.32	0.134
	iCCM-ready HSA density (per 1,000 under-five children)	1.17	2.18	0.596
Change in mortality rate between baseline and endline (deaths per 1,000 live births)	Intercept*	−37.03	18.30	0.055
	District population (total population/100,000)	1.16	2.25	0.610
	Health facility density (per 10,000 total population)	13.06	21.48	0.550
	Facility worker density (per 10,000 total population)	1.19	1.28	0.363
	iCCM-ready HSA density (per 1,000 under-five children)	−3.07	8.82	0.731

HSA = health surveillance assistants; iCCM = integrated Community Case Management; OLS = ordinary least square; SE = standard error.

* Intercept interpretable as the expected change for average district population (426,300), with a facility and facility worker density of zero and an iCCM-ready HSA density of zero.

**Table 4:** Estimated recurring and annualized capital costs of iCCM program, 2012 U.S. Dollars (95% confidence interval)

Variable	Training, supervision	Other program costs	Salaries	Equipment	Drugs	Total
Cost per HSA	$154 ($106–211)	$169 ($139–198)	$503 ($390–603)	$39 ($35–42)	$947 ($656–1,250)	$1,812 ($1,327–2,304)
Cost per case seen	$0.16 ($0.11–0.22)	$0.17 ($0.14–0.20)	$0.52 ($0.40–0.62)	$0.040 ($0.036–0.044)	$0.97 ($0.67–1.28)	$1.86 ($1.36–2.37)
Cost per district	$20,335 ($14,057–27,943)	$22,435 ($18,469–26,176)	$66,651 ($51,711–79,863)	$5,120 ($4,619–5,620)	$125,481 ($86,892–165,524)	$240,022 ($175,748–305,128)
Cost per child U5 in district*	$0.21 ($0.14–0.28)	$0.23 ($0.19–0.26)	$0.67 ($0.52–0.81)	$0.052 ($0.047–0.057)	$1.27 ($0.88–1.67)	$2.43 ($1.78–3.09)
Estimated cost of iCCM program (millions)†	$0.59 ($0.41–0.81)	$0.65 ($0.54–0.76)	$1.93 ($1.50–2.32)	$0.15 ($0.13–0.16)	$3.64 ($2.52–4.80)	$6.96 ($5.10–8.85)

* All children in district (not just children living in areas served by HSAs).

† Estimate for the entire country, based on the number of HSAs trained on iCCM in the country.
