# Supplementary material for: Independent Evaluation of the integrated Community Case Management of Childhood Illness Strategy in Malawi Using a National Evaluation Platform Design
Source: Am J Trop Med Hyg. 2016 Mar 2;94(3):574–83. doi: 10.4269/ajtmh.15-0584 (PMC4775894; doi:10.4269/ajtmh.15-0584)
Supplement: Supplementary file 1 [file SD6.pdf]

SUPPLEMENTAL WEB ANNEX: STATISTICAL METHODS AND ADDITIONAL RESULTS

Part 1: Development of summary measures of integrated Community Case Management implementation strength. As part of our primary analysis, we ran factor analysis on the implementation strength snapshot (ISS) data set containing the six "core" indicators of integrated Community Case Management (iCCM) implementation strength as defined by the Malawi Ministry of Health (MOH) and iCCM stakeholders. We extracted the first component for use as our underlying or latent variable. The distribution of these indicators is shown in Supplemental Table 1.1, and their correlations are shown in Supplemental Figure 1.1.

As shown in Supplemental Table 1.1, three of the six indicator frequencies were very close to 100% (health surveillance assistant [HSA] trained, HSA deployed, and HSA received initial iCCM drug kit), and thus were not informative about differences in implementation strength between HSAs or districts.

We used the correlation matrix to estimate the implementation strength for each HSA.[1] However, a simple additive score of the readiness of each HSA was closely related to this factor-analyzed implementation strength, with a correlation for all HSAs in the ISS survey of 0.94.

We therefore moved forward with a simple additive score of the readiness of each HSA to provide iCCM, based on whether they had been supervised in the past 3 months, whether the most recent supervision visit included the observation of clinical practice, and whether there had been no stockouts in the previous 3 months. These indicators are not independent of each other, and in general are closely related. Their sum produced a readiness score with a range of 0–3. This score was strongly correlated (r = 0.94) with the more complex score derived through factor analysis. An HSA was considered to be "iCCM-ready" if they had a score of 2–3.

Supplemental Table 1.1
Summary of implementation indicators for HSAs from the ISS survey

Implementation indicator	Percent of HSAs
Trained in CCM	99
CHW deployed and working	100
Received initial drug kit	99
Supervised/mentored in past 3 months	60
Supervised in past 3 months with clinical feedback or mentored	58
No stockout of CCM drug in 3 months	38

CCM = community case management; CHW = community health worker; HSA = health surveillance assistants; ISS = implementation strength snapshot.

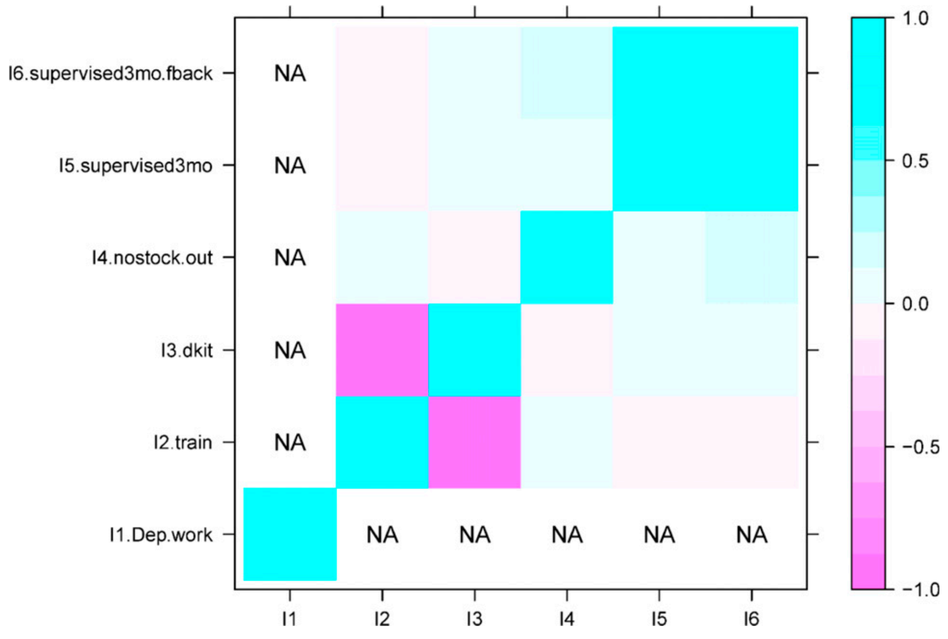

Supplemental Figure 1.1. Correlation matrix of implementation strength indicators for 3,392 health surveillance assistants from the implementation strength snapshot survey.

Part 2: Results on careseeking for individual diseases (pneumonia, diarrhea, and malaria) and by type of provider. In the main analyses, we report on a careseeking indicator for any child with diarrhea, pneumonia, or fever/malaria. In this section, we report on findings for each condition separately. Supplemental Table 2.1 shows the reported number of illness episodes in the previous 2 weeks by disease, as well as their co-occurrence. The considerable overlap among diseases is shown in a Venn diagram in Supplemental Figure 2.1.

Supplemental Table 2.2 shows the prevalence of careseeking (%) from any provider and from HSAs for children reporting each of the three diseases.

Supplemental Table 2.3 shows the intercorrelations between changes in careseeking for the three diseases between 2010 and 2014, showing some evidence of moderate positive associations. The correlation was strongest for changes in careseeking due to malaria and pneumonia.

Supplemental Table 2.4 shows careseeking for each of the three iCCM illnesses (fever, diarrhea, and pneumonia) by careseeking source for the baseline (DHS 2010) and endline (MDG 2014).

Supplemental Table 2.1

Reported illness episodes in previous 2 weeks, 2014 MDG survey

	Pneumonia		No pneumonia		
Counts	Diarrhea	No diarrhea	Diarrhea	No diarrhea	Total
Fever	290	576	1,857	4,395	7,118
No fever	151	421	2,121	9,170	11,863
Total	441	997	3,978	13,565	18,981

MDG = Millennium Development Goal.

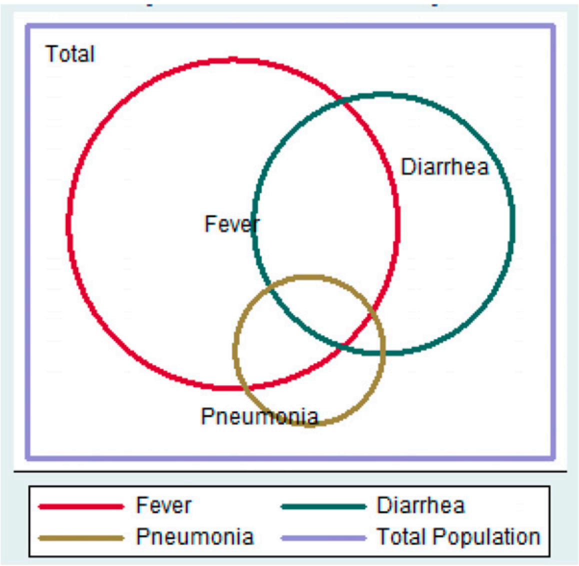

Supplemental Figure 2.1. Venn diagram showing the overlap of diarrhea, pneumonia, and fever in the 2014 Millennium Development Goal survey.

Supplemental Table 2.2

Unweighted, descriptive district results (%) on careseeking for children with symptoms of pneumonia, diarrhea, and malaria and year in which data were collected in 27 districts in Malawi

District variables	Year	Mean (%)	Median (%)	Minimum (%)	Maximum (%)
Pneumonia: any formal provider					
Baseline careseeking	2010	75.0	76.4	36.7	96.8
Endline careseeking	2014	69.7	70.9	47.5	89.9
Change in careseeking between baseline and endline	–	−5.3	−5.5	10.8	−6.9
Pneumonia: HSAs					
Baseline careseeking	2010	1.3	0.0	0.0	5.3
Endline careseeking	2014	8.0	7.0	0.0	24.9
Change in careseeking between baseline and endline	–	6.7	7.0	0.0	19.6
Diarrhea: any formal provider					
Baseline careseeking	2010	66.7	64.1	53.4	79.6
Endline careseeking	2014	69.3	69.0	58.1	81.0
Change in careseeking between baseline and endline	–	2.6	4.9	4.7	1.4
Diarrhea: HSAs					
Baseline careseeking	2010	3.1	2.7	0.0	10.9
Endline careseeking	2014	11.3	9.5	2.6	27.0
Change in careseeking between baseline and endline	–	8.2	6.8	2.6	16.1
Malaria: any formal provider					
Baseline careseeking	2010	68.4	68.0	56.4	79.8
Endline careseeking	2014	67.1	67.1	52.0	79.6
Change in careseeking between baseline and endline	–	−1.3	−0.9	−4.4	−0.2
Malaria: HSAs					
Baseline careseeking	2010	1.6	1.1	0.0	5.3
Endline careseeking	2014	8.8	7.7	0.9	20.4
Change in careseeking between baseline and endline	–	7.2	6.6	0.9	15.1

HSA = health surveillance assistant.

Supplemental Table 2.3

Correlations between changes in careseeking for pneumonia, diarrhea, and malaria from baseline 2010 DHS to endline 2013 MDG survey in 27 districts in Malawi

	Endline–baseline careseeking for pneumonia	Endline–baseline careseeking for diarrhea	Endline–baseline careseeking for malaria
Endline-baseline careseeking for pneumonia	1.00	0.29 (*P* = 0.136)	0.49 (*P* = 0.009)
Endline-baseline in careseeking for diarrhea	0.29 (*P* = 0.136)	1.00	0.38 (*P* = 0.049)
Endline-baseline careseeking for malaria	0.49 (*P* = 0.009)	0.38 (*P* = 0.049)	1.00

DHS = Demographic and Health Surveys; MDG = Millennium Development Goal.

Supplemental Table 2.4

Careseeking for the three iCCM illnesses (fever, diarrhea, and pneumonia) by careseeking source for the baseline (DHS 2010) and endline (MDG 2014)

	Baseline (DHS 2010) (%)	Endline (MDG 2014) (%)
Appropriate careseeking	67.6	68.1
Public health sector (including HSA)	54.2	60.8
Public health sector (excluding HSA)	52.3	51.2
HSA	2.6	10.4
Private health sector* (excluding pharmacies)	15.2	7.8
Informal sector (pharmacies, shops, and friends/neighbors)	11.3	14.2
No careseeking	30.2	26.6

DHS = Demographic and Health Surveys; HSA = health surveillance assistant; iCCM = integrated Community Case Management; MDG = Millennium Development Goals. These are individual averages, not district averages as shown in the article.

*Mission hospitals/clinics (Christian Health Association of Malawi (CHAM)) are included under private health sector.

Part 3: Analyses for under-five mortality and careseeking. Supplemental Table 3.1 shows the OLS (unweighted) of the changes in careseeking for childhood illness and under-five mortality on iCCM implementation strength, controlling for contextual factors.

Supplemental Table 3.1

Ordinary unweighted least squares linear regression of the changes in careseeking for childhood illness and under-five mortality, predicted by iCCM implementation strength and controlling for contextual factors

Outcome	Predictors	Estimate	SE	*P*
Change in careseeking between baseline and endline (% points)	Intercept*	7.70	6.10	0.220
	District population (total population/100,000)	−0.26	0.55	0.643
	Health facility density (per 10,000 total population)	−3.47	5.22	0.513
	Facility worker density (per 10,000 total population)	−0.48	0.31	0.140
	Ready HSA density (per 1,000 under-five children)	1.30	2.15	0.550
Change in mortality rate between baseline and endline (deaths per 1,000 live births)	Intercept*	−36.56	22.44	0.117
	District population (total population/100,000)	−1.07	2.76	0.701
	Health facility density (per 10,000 total population)	5.89	26.33	0.825
	Facility worker density (per 10,000 total population)	0.67	1.57	0.674
	Ready HSA density (per 1,000 under-five children)	−4.55	10.82	0.678

HSA = health surveillance assistant; iCCM = integrated Community Case Management; SE = standard error.
*Intercept interpretable as the expected change for average district population (426,300), with a facility and facility worker density of zero and an iCCM-ready HSA density of zero.

Part 4: Two-stage least squares regression methods and results. We used an additional approach to the dose–response analyses based on two-stage least squares (TSLS) regression. Economists have been using this approach for many years to understand causal effects in observed data.[2,3] If implementation strength were randomly assigned to each district, then OLS regression would yield unbiased estimates of the effect of iCCM on changes in mortality and careseeking, by assuming

$$Y = \beta_0 + \beta_1 X_1 + \rho \mathrm{IS} + \grave{o}$$

where Y is the outcome, X_1 are contextual factors, IS is implementation strength, E is a normally distributed error, and ρ is the primary relationship of interest. However, implementation strength was not random, instead it was constrained in part by factors particular to each district. If we can identify factors X_2 that are associated with implementation strength, but are not directly related to the change in mortality or careseeking except through implementation, implementation strength can be predicted by

$$\mathrm{IS} = \gamma_0 + \gamma_1 X_1 + \gamma_2 X_2 + \eta$$

Then, we use an additional second stage regression using the predicted implementation strength, whose effect ρ has a causal interpretation under certain conditions. The second stage regression takes the form

$$Y = \beta_0 + \beta_1 X_1 + \rho \widehat{\mathrm{IS}} + \grave{o}^*$$

where E^* is an error term distinct from E. For this analysis to be valid, three conditions must hold: 1) factor X_2 is related to implementation strength but not directly to the change in mortality or careseeking, 2) relationships between X_1, X_2 and implementation strength allow for uniquely identified effects, and 3) factor X_2 is predictive of implementation strength.[3] A summary of these conditions is shown in Table 2 for implementation strength predicted by district population, health facility density, and facility worker density.

A summary of results for TSLS is shown in Supplemental Table 4.1.

Supplemental Table 4.1

Summary of results for TSLS regression models for the change in U5MR and careseeking for pneumonia, diarrhea, and malaria in 27 districts in Malawi

Outcome	Predictors	Estimate	SE	*P*
Endline–baseline U5MR	Intercept*	−34.33	15.71	0.029
	District population	−1.27	2.61	0.627
	Predicted iCCM-ready HSA density†	7.07	18.28	0.699
Endline–baseline careseeking	Intercept*	−1.71	2.87	0.551
	District population	0.34	0.57	0.554
	Predicted iCCM-ready HSA density†	1.67	3.51	0.634

HSA = health surveillance assistant; iCCM = integrated Community Case Management; SE = standard error; TSLS = two-stage least squares; U5MR = under-five mortality rate.
*Intercept interpretable at average district population (426,300).
†Predicted by health facilities and facility worker density.

Part 5: Results for baseline mortality and careseeking. In the main analysis, we accounted for baseline levels of the outcomes by incorporating changes in the levels of the outcome variables (careseeking, mortality) as change variables (endline minus baseline levels). We also used the endline value as the dependent variable, including the baseline value as one of the independent variables. These results are presented in Supplemental Table 5.1.

Supplemental Table 5.1

OLS regression of endline U5MR and careseeking, predicted by implementation strength and contextual factors, including baseline mortality and careseeking

Outcome	Predictors	Estimate	SE	*P*
Endline U5MR	Intercept*	76.39	19.71	0.001
	District population	−2.23	6.00	0.971
	Baseline U5MR	−0.04	0.13	0.764
	Health facilities	−4.46	14.78	0.761
	Facility worker density	−0.50	1.48	0.740
	Ready HSA density	4.44	6.52	0.504
Endline careseeking	Intercept*	45.9	6.10	0.220
	District population	−0.26	0.21	0.226
	Baseline careseeking	0.41	0.23	0.087
	Health facilities	−4.95	5.06	0.339
	Facility worker density	0.53	0.57	0.363
	Ready HSA density	−0.95	2.21	0.672

HSA = health surveillance assistant; OLS = ordinary least squares; SE = standard error; U5MR = under-five mortality rate.

*Intercept interpretable as the expected endline measure for average district population (426,300), with a facility and facility worker density of zero and an integrated Community Case Management–ready HSA density of zero

Part 6: Correlation matrix.

Supplemental Table 6.1
Correlation matrix

		1	2	3	4	5	6	7	8	9	10	11	12	13	14	15
1	Under-five population (in thousands)	1.00														
2	Poverty (%)	−0.31	1.00													
3	Any maternal education (% of mothers)	−0.11	−0.53	1.00												
4	Health facility worker density (per 10,000 total population)	−0.59	0.21	0.21	1.00											
5	Proportion of women reporting that distance to health facility is a problem	−0.25	0.09	−0.18	0.15	1.00										
6	Density of HSAs working iCCM (per 1,000 under-five children	−0.33	−0.04	0.09	0.43	0.01	1.00									
7	iCCM readiness	0.12	−0.20	0.00	−0.16	−0.03	−0.07	1.00								
8	Children treated by HSAs (per 10,000 under-five children per month)	−0.45	−0.14	0.13	0.45	0.08	0.77	0.19	1.00							
9	Density of HSAs with high iCCM readiness (per 1,000 under-five children)	−0.29	−0.01	−0.03	0.37	0.09	0.85	0.38	0.78	1.00						
10	Baseline U5MR (2007–2009)	0.18	0.05	−0.30	−0.20	0.10	0.14	0.16	−0.09	0.11	1.00					
11	Baseline careseeking for iCCM conditions	−0.45	0.11	0.49	0.69	−0.21	0.30	−0.21	0.36	0.15	−0.28	1.00				
12	Baseline careseeking from HSA for iCCM conditions	−0.17	0.13	0.01	−0.13	0.11	0.06	−0.17	−0.09	−0.06	0.36	−0.02	1.00			
13	Change in U5MR between baseline and endline	−0.12	−0.10	0.24	0.12	−0.13	0.02	−0.20	0.08	−0.03	−0.86	0.20	−0.43	1.00		
14	Change in careseeking for iCCM conditions between baseline and endline	0.01	−0.40	−0.10	−0.20	0.44	−0.20	0.25	−0.01	0.02	0.11	−0.49	0.04	−0.19	1.00	
15	Change in careseeking from HSA for iCCM conditions between baseline and endline	−0.04	−0.17	0.03	−0.20	0.32	0.19	0.19	0.15	0.24	0.27	−0.22	0.18	−0.36	0.22	1.00

HSA = health surveillance assistant; iCCM = integrated Community Case Management; U5MR = under-five mortality rate.
Significance levels are shown by shading, with $P < 0.05$ in dark gray shading and $P < 0.10$ in light gray shading.

Part 7: Additional dose–response results. Correlations between the density of iCCM-ready HSAs and changes between 2010 and 2014 in careseeking for childhood illness and mortality rates in children under 5 years of age, for 27 districts in Malawi. (Supplemental Figure 7.1).

Correlations between the two component parts of the iCCM implementation readiness (the density of HSAs per 1,000 under-five children and average district iCCM-readiness scores) and changes between 2010 and 2014 in outcomes measures (careseeking for childhood illness and under-five mortality rate). (Supplemental Figure 7.1).

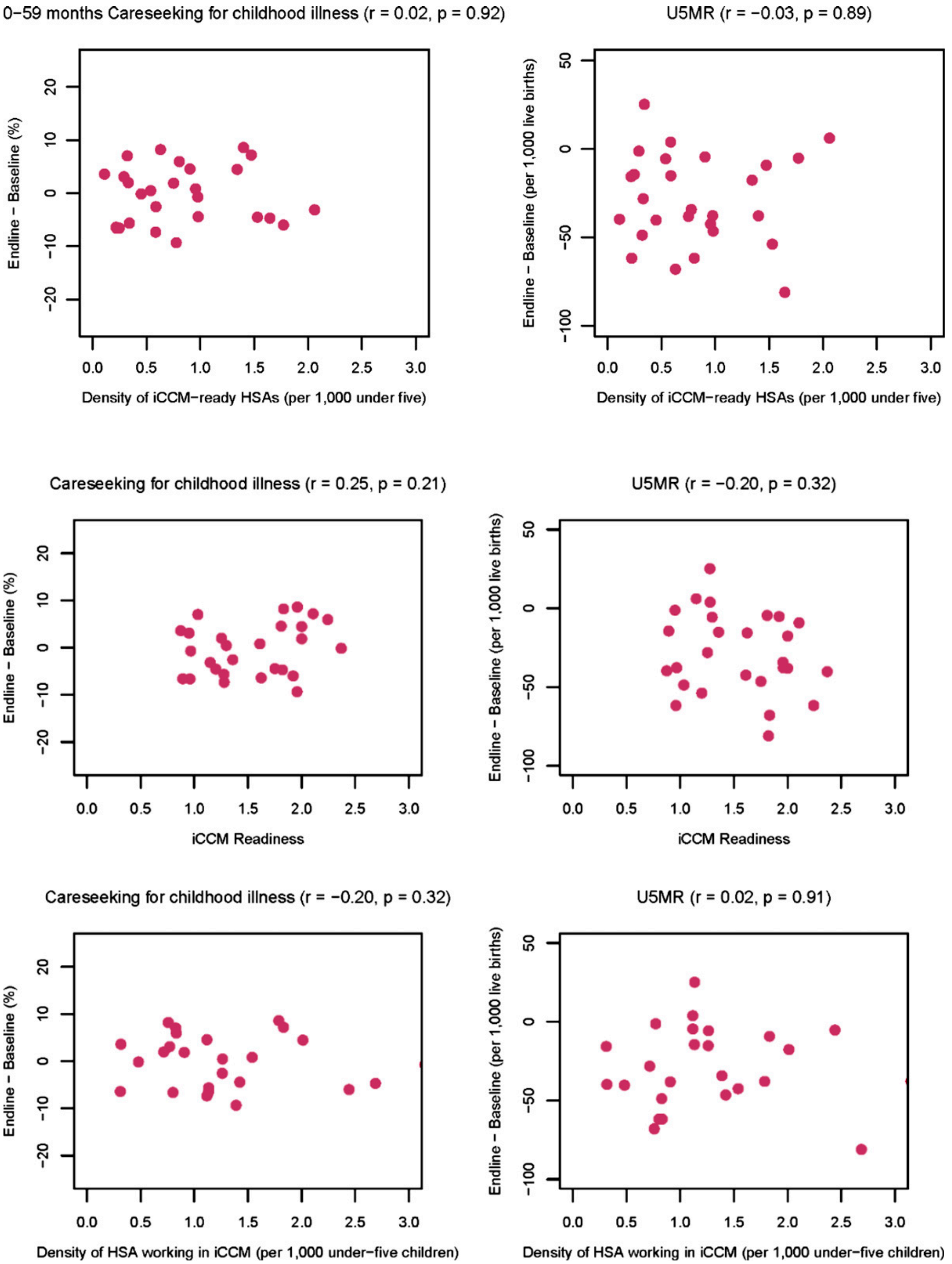

SUPPLEMENTAL FIGURE 7.1. Correlation between change in careseeking, under-five mortality rate and measures of implementation strength.

Part 8: Careseeking by wealth quintile. Supplemental Figure 8.1 presents levels of careseeking at baseline and endline by type of provider, and according to wealth quintile.

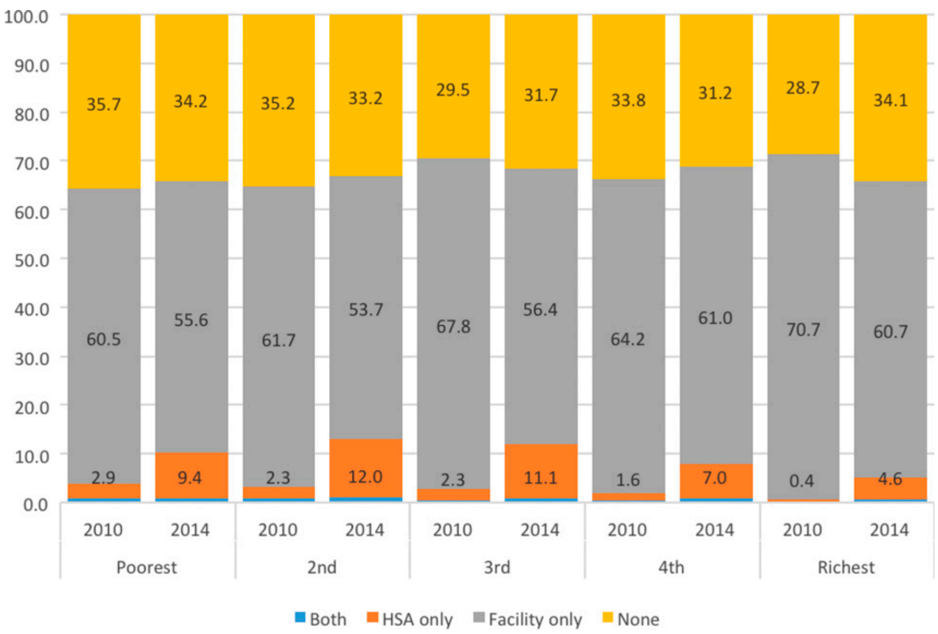

Supplemental Figure 8.1. Careseeking at baseline (2010) and endline (2014) by type of provider, according to wealth quintile.

Part 9: Details of costing methods. Data were collected for calendar year 2012. We collected data from six districts in Malawi, of which four districts were in the central region and two were in the southern region. Four of these districts were selected based on inclusion in a baseline costing study of child health services in Malawi. The additional two districts were selected in discussion with the MOH to increase the representativeness of the results, subject to available study resources (the additional districts selected are located in the northern part of the central region).

Three research teams attended a weeklong training prior to data collection. Each team consisted of one supervisor and five data collectors. Wherever possible, we selected supervisors from among district health office (DHO) staff in each of our districts to facilitate data collection activities. A staff member from Institute for International Programs (IIP) or Chancellor College accompanied each team during data collection. The main phase of data collection occurred over a period of 6 weeks from August to September 2013. Data were double-entered into Microsoft Excel (Arlington, VA) and checked by study staff prior to analysis.

In each district, we collected the following cost data at the HSA, health facility, DHO, and implementing partner levels:

- HSA data from HSAs affiliated with six health facilities included two assessments of their time use, a sample of 24 records from the HSA patient register, and an assessment of equipment and supplies provided to HSAs for iCCM. Details of the methods for the assessment of HSA time use have been presented elsewhere.[4]
- Health facility data from six health facilities included drugs, medical supplies, and non-medical supplies distributed to HSAs and iCCM utilization data for HSAs within the health facility catchment area.
- DHO data included HSA salaries, including any monetary or nonmonetary incentives provided to HSAs. DHO data also included district-specific program costs paid by the government to support iCCM, though these were generally difficult to estimate and have not been included.
- Implementing partner data included program costs associated with supporting iCCM nationally and at the district level. National program costs were allocated to district costs based on the number of districts in which the partner operated. Program costs included supervision, mentorship, sensitization, promotion/communication materials, and training. Training included initial iCCM training, refresher training, supervision training, and any other training. With the exception of training costs, all program costs were limited to calendar year 2012. Training costs were collected for the period 2009–2012. Implementing partner data also included overhead costs, such as partner staff costs and equipment.

Data collection also included demographic, programmatic, and price components at the national level. We collected staffing levels for HSAs trained in iCCM from the MOH. We used the population projections from the 2008 Census to estimate the population of children under 5 years of age in each district.[5] We collected national drug and commodity price data from the Central Medical Stores (CMS) catalog whenever possible. These data were occasionally unavailable through CMS, in which case price data was collected from local sources.

The study protocol was submitted and approved by the institutional review boards of Johns Hopkins Bloomberg School of Public Health and the National Commission for Science and Technology of Malawi.

Data analysis. Program costs. The cost of training an HSA on iCCM was estimated assuming that the training would last for 5 years, on average, while refresher training was assumed to be needed every 4 years. Refresher training, supervision, and staff and equipment costs above the level of service delivery were assumed to be needed on an annual basis. For these latter costs, we calculated the total costs of all activities in 2012 and divided it by the number of active HSAs delivering iCCM to determine the cost per HSA per year.

Drug costs. Average drug costs per child seen were computed based on a sample of patient records taken from HSA registers. Missing values were estimated using multiple imputation with chained equations. The total cost per case was estimated as the sum of the individual drug costs, which were calculated as the product of the quantities and prices. Average costs per case and 95% confidence intervals (CIs) were calculated at the district and national levels. Uncertainty due to imputation of missing data accounted for less than 2% of the overall uncertainty.

Salaries. To determine the average salary of HSAs, we used the weighted average of salaries and benefits based on distribution of HSAs among different pay grades. We used the average proportion of time HSAs spent on iCCM to estimate the salary and benefit costs of HSAs.

Equipment. We took an inventory of equipment in possession of HSAs at the facilities visited. We then estimated the average annual costs of this equipment for an HSA.

Annualization. Cost was annualized using a 3% discount rate. Start-up activities, such as training and community sensitization, were assumed to have useful life of 4–5 years on average. Capital items, such as equipment, were annualized based on the estimated useful life of each item.

Uncertainty analysis. The following table provides the mean estimate for each input variable, the type of distribution used around the mean, and the parameters for the distribution method for the Monte Carlo analysis. From each distribution, we drew 1,000 values at random, sorted the results in order of value, and used the 25th and 975th observations as the 95% CI.

Supplemental Table 9.1

Mean estimate for each input variable, the type of distribution used around the mean, and the parameters for the distribution method for the Monte-Carlo analysis

Cost variable	Mean per HSA per year	Distribution	Distribution parameters
Costs above service delivery level			
Initial training of HSAs on iCCM	US$87.44	Normal	SE = 18.2
Refresher training	US$21.55	Truncated normal	SE = 21.3
Supervision	US$25.88	Normal	SE = 2.5
Other training	US$18.66	Normal	SE = 2.1
Sensitization	US$1.28	Truncated normal	SE = 1.2
Programmatic staff	US$159.17	Normal	SE = 15.4
Equipment for programmatic staff	US$8.93	Normal	SE = 0.7
Costs at service delivery level			
Salaries/benefits of HSA time spent on iCCM	US$503.23	Uniform	Range = 390.4–603.0
HSA equipment	US$38.65	Truncated normal	SE = 1.5
Drugs	US$434.32	Multivariate normal	SE for cost per case = 0.02; SE for number of cases seen = 145.9

HSA = health surveillance assistants; iCCM = integrated Community Case Management; SE = standard error.
Values are rounded for ease of presentation.

Supplemental Table 9.2

Estimated recurring and annualized capital costs of CCM program, 2012 U.S. dollars (95% CI)

Variable	Training, supervision	Other program costs	Salaries	Equipment	Drugs	Total
Cost per HSA (US$)	154 (106–211)	169 (139–198)	503 (390–603)	39 (35–42)	947 (656–1,250)	1,812 (1,327–2,304)
Cost per case seen (US$)	0.16 (0.11–0.22)	0.17 (0.14–0.20)	0.52 (0.40–0.62)	0.040 (0.036–0.044)	0.97 (0.67–1.28)	1.86 (1.36–2.37)
Cost per district (US$)	20,335 (14,057–27,943)	22,435 (18,469–26,176)	66,651 (51,711–79,863)	5,120 (4,619–5,620)	125,481 (86,892–165,524)	240,022 (175,748–305,128)
Cost per child under 5 years of age in district* (US$)	0.21 (0.14–0.28)	0.23 (0.19–0.26)	0.67 (0.52–0.81)	0.052 (0.047–0.057)	1.27 (0.88–1.67)	2.43 (1.78–3.09)
Estimated cost of CCM program (millions)† (US$)	0.59 (0.41–0.81)	0.65 (0.54–0.76)	1.93 (1.50–2.32)	0.15 (0.13–0.16)	3.64 (2.52–4.80)	6.96 (5.10–8.85)

CCM = community case management; CI = confidence interval; HSA = health surveillance assistant.
* All children in district (not just children living in areas served by HSAs).
†Estimate for the entire country, based on the number of HSAs trained on integrated Community Case Management in the country.

SUPPLEMENTAL REFERENCES

1. Knott M, Bartholomew DJ, 1999. *Latent Variable Models and Factor Analysis*. New York, NY: Arnold Publishers.
2. Durbin J, 1954. Errors in variables. *Rev Int Stat Inst 22:* 23–32.
3. Angrist JD, Imbens GW, 1995. Two-stage least squares estimation of average causal effects in models with variable treatment intensity. *J Am Stat Assoc 90:* 431–442.
4. Carter E, Marsh A, Munthali S, Chirwa G, Walker N, Johns B, Amouzou A, 2014. *Community Health Worker Time Use: Method, Evaluation and Findings in Malawi*. Poster Presentation at the Annual Meeting of the American Public Health Association, New Orleans, LA., May 1–3, 2014, Boston, MA.
5. National Statistical Office, Malawi, 2008. *2008 Population and Housing Census, Preliminary Report*. Available at: http://www.mw.one.un.org/wp-content/uploads/2014/04/Malawi-Population-and-Housing-Census-Preliminary-Report-2008.pdf. Accessed December 9, 2014.
